# Editorial: Current chemical approaches in combating neuroinflammation in Alzheimer's disease (AD)

**DOI:** 10.3389/fnagi.2025.1712601

**Published:** 2025-10-22

**Authors:** Sayan Roy Chowdhury, Venkatesh Katari, Dwaipayan Sen, Prasenjit Mondal

**Affiliations:** ^1^Department of Biochemistry and Molecular Biophysics, Washington University School of Medicine, Saint Louis, MO, United States; ^2^Department of Physiology and Pharmacology, College of Medicine and Life Sciences, The University of Toledo, Toledo, OH, United States; ^3^Innovative Medicines Accelerator, Stanford University, Stanford, CA, United States; ^4^Genetics and Aging Research Unit, McCance Center for Brain Health, Mass General Institute for Neurodegenerative Disease, Department of Neurology, Massachusetts General Hospital, Harvard Medical School, Charlestown, MA, United States

**Keywords:** NLRP3 inflammasome, amyloid-beta, tauopathies, Alzheimer's disease, inflammation, TREM family, cognition

**Alzheimer's disease (AD)**, which is projected to affect over 80 million individuals globally by 2040, is still the most prevalent form of dementia and a serious public health concern (Alzheimer's Association, 2023). The extracellular deposition of amyloid-β (Aβ) plaques and intraneuronal tangles of hyperphosphorylated tau have historically been used to describe the disease (Bloom, 2014). Neuroinflammation has drawn special attention among the new discoveries as a crucial and early factor in the development and progression of the disease. Neuroinflammatory processes seem to precede and intensify amyloid and tau pathology, rather than being a downstream effect of it (Leng and Edison, 2021). This accelerates synaptic dysfunction, neuronal death, and cognitive decline, indicating the need for novel pharmacological intervention that explicitly target inflammatory pathways in AD (Li et al., 2018; Gennaccaro et al., 2021). In addition to the development of biomarkers, strategies for the development of therapeutics are needed. Cerebrospinal fluid proteins like macrophage migration inhibitory factor (MIF), soluble TREM1 and TREM2 (triggering receptor expressed on myeloid cells), are implicated in the dynamic stages of AD development, often correlating with tau pathology and neuroinflammation, rather than with amyloid deposition/aggregation (Bonomi et al., 2023). Further, imaging studies using positron emission tomography (PET) tracers directed against the translocator protein (TSPO), indicate that microglial activation (a hallmark of neuroinflammation in AD) is associated with future cognitive decline independent of amyloid or tau load (Vivash and O'Brien, 2016).

The interaction between cerebral disease and peripheral immunological processes is further highlighted by systemic indicators of inflammation, lipid profiles, and vascular dysfunction. When taken as a whole, these biomarker developments not only shed light on molecular pathways but also present the opportunity to monitor therapy efficacy and stratify patients for early intervention (Zhu et al., 2024).

Concurrently, the chemical approaches intended to alter neuroinflammatory pathways has advanced significantly. One of the most promising approaches is inflammasome inhibition, specifically targeting (NLR family pyrin domain containing 3 protein) NLRP3 inflammatory pathways (Grebe et al., 2018; Heneka et al., 2024). Structure-based designs have produced multifunctional small compounds that can reduce microglial activation, cross the blood–brain barrier, and improve behavioral abnormalities in transgenic animal models (Chen et al., 2022). In addition to *de novo* medication discovery, repurposing metabolic agents has yielded promising outcomes. Liraglutide, a GLP-1 receptor agonist that was first created to treat diabetes and obesity, dramatically decreased brain atrophy in areas of the brain that are important for memory and learning in individuals with moderate AD in a recent trial (Anderer, 2025). This finding suggests that the medication may have neuroprotective and anti-inflammatory properties. Collectively, these studies demonstrate the viability of structure-guided and receptor-based pharmacological approaches to reduce neuroinflammation.

Furthermore, inflammation in the brain is not uniformly deleterious. Microglia and astrocytes play dual roles, at times promoting clearance of toxic proteins and supporting neuronal health, and at other times exacerbating injury through chronic activation (Grebe et al., 2018). Broad suppression of inflammatory responses may therefore be counterproductive, highlighting the need for strategies that modulate rather than abolish these pathways. Additional obstacles include the perennial difficulties of drug delivery across the blood–brain barrier, achieving adequate potency without off-target toxicity, and the frequent exclusion of neuroinflammatory markers as primary outcomes in clinical trials.

Rational molecular design acquainted by receptor structures and detailed structure-activity relationship studies will be essential for optimizing potency, brain penetrance, and safety. Ultimately, the success of chemical approaches will likely require combination strategies. Anti-inflammatory molecules may synergize with amyloid- and tau-directed agents, as well as with metabolic and vascular interventions, to achieve durable disease modification. Clinical trial designs must evolve to accommodate this complexity, incorporating multimodal biomarkers, robust surrogate endpoints, and longer observation periods to detect meaningful changes.

**Figure 1:**
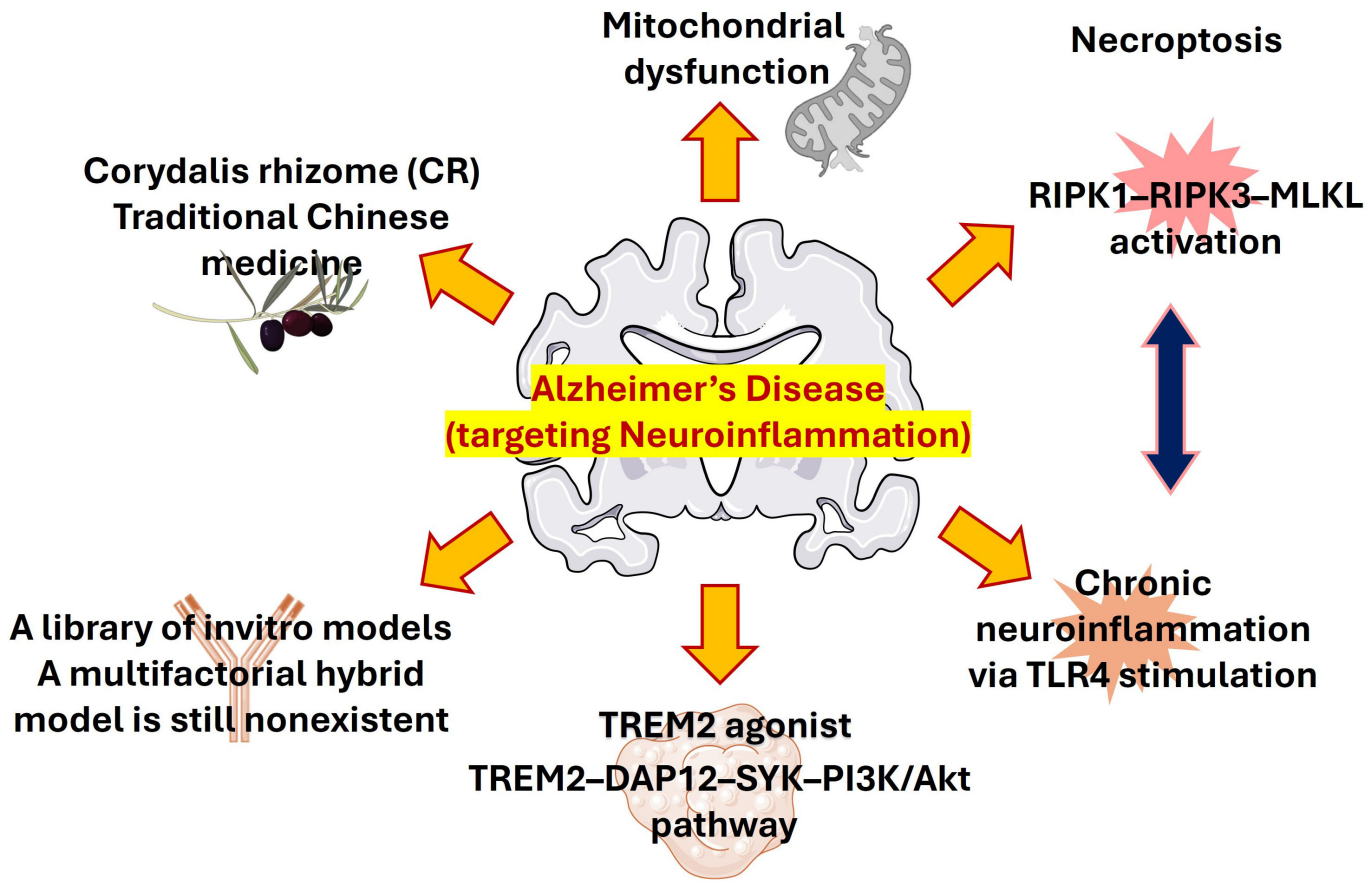
Schematic showing approaches to target neuroinflammation in Alzheimer's disease.

Chemical approaches that helped in studying AD models are still an essential tool for understanding the disease mechanism and may aid in the evaluation of newly designed compounds. Selective AD-like behaviors are reproduced by agents such heavy metals, okadaic acid, Prostaglandin J2 (PGJ2), Aβ, scopolamine, and lipopolysaccharide (LPS) through different signaling pathways (Figueiredo-Pereira et al., 2016; Mondal et al., 2024, 2018). Finding similar molecular processes is crucial for logical model selection and trustworthy drug evaluation, even when each model emphasizes distinct clinical stages. Here we highlight several studies of chemical strategies targeting neuroinflammatory pathways to ameliorates AD pathological symptoms. A schematic representation of the overall approaches targeting neuroinflammation in AD have shown in Figure 1.

The medicinal potential of Corydalis rhizome (CR), a traditional Chinese medication enhanced with protoberberine-type alkaloids, is being highlighted by Lyu et al. CR may have combinatorial benefits through anti-inflammatory, antioxidant, and neurotransmitter-modulating mechanisms, improving memory and anxiety-like behaviors in AD rats. Its capacity to change the phenotype of microglia from M1 to M2 via IL-6/JAK2/STAT3 signaling highlights a potentially effective natural anti-inflammatory treatment.

Basheer et al. have described the chronic stimulation of TLR4 in tau-transgenic mice enhances microglial activation without influencing tau tangle formation. This finding illustrates the intricate interplay between neuroinflammation and tau pathology, suggesting that inflammation alone may be insufficient to drive tau aggregation. Understanding this dissociation will be crucial for refining neuroinflammation-targeted therapies in AD.

The study by Wei et al., described the dysfunction of mitochondria that plays an important role in AD pathogenesis, and in addition, recent studies also revealed the dynamic intercellular mitochondrial transfer between neurons and glial cells. This review concisely described the exciting phenomenon of mitochondrial transfer that offers an exciting frontier for disentanglement AD mechanisms and therapeutic innovation.

This topic not only discussed about the chemical approaches developed for combating neuroinflammation but also discussed why certain drugs developed for a promising target has failed in clinical translation. Recently, a microglial receptor protein TREM2, gained a lot of interest due to its promising role in clearing of pathological substrates. Ma et al. have discussed the failure of the INVOKE-2 trial with the TREM2 antibody AL002 highlighting the challenges in clinical translation in mini review.

Additionally, exercise, a non-pharmacological approach has also emerged as a powerful regulator of necroptosis and neuroinflammation. Lu et al. demonstrated exercise as a potent neuroprotective in AD as it can significantly reduce various inflammatory cytokines like TNF-α, HMGB1, and IL-1β, and improve O-GlcNAc glycosylation to limit tau hyperphosphorylation. These insights clearly strengthen the rationale for integrating lifestyle interventions in preventing AD progression.
